# How Mind-Body Practice Works—Integration or Separation?

**DOI:** 10.3389/fpsyg.2017.00866

**Published:** 2017-05-26

**Authors:** Yi-Yuan Tang, Changhao Jiang, Rongxiang Tang

**Affiliations:** ^1^Department of Psychological Sciences, Texas Tech UniversityLubbock, TX, United States; ^2^Beijing Key Lab of Physical Fitness Evaluation and Tech Analysis, Capital University of Physical Education and SportsBeijing, China; ^3^Department of Psychological and Brain Sciences, Washington University in St. LouisSt. Louis, MO, United States

**Keywords:** mind-body practice, mindfulness meditation, integrative body–mind training (IBMT), MBSR, MBCT, central nervous system, autonomic nervous system

In this *Opinion* piece, we take mindfulness as an example of mind-body practice to explore the question of how mind-body practice works based on recent neuroimaging evidence. Mind-body practice encompasses a family of complex practices such as mindfulness meditation, Tai Chi, Yoga, and Qi Gong. Of these practices, mindfulness meditation has received the most attention in the field of psychology and neuroscience over the past two decades. In our recent review, we summarize that mindfulness meditation includes three components that interact closely to constitute a process of enhanced self-regulation: enhanced attention control, improved emotion regulation and altered self-awareness. We also pointed out many people use the term “mindfulness,” but often refer to completely different things or ideas (Tang et al., 2015). In the following sections, we provide examples to briefly clarify the fundamental understanding of the mindfulness concept and how mind-body practice works.

## The clarification of mindfulness

One recent perspective divides mindfulness meditation or intervention into (1) “mindfulness-based stress reduction (MBSR) and related group-based mindfulness interventions such as mindfulness-based cognitive therapy (MBCT), and (2) mindfulness-related interventions such as acceptance and commitment therapy (ACT), dialectical behavior therapy (DBT), cognitive behavioral stress management, and integrative body-mind training (IBMT)” (Creswell, 2017). The confusion is that the phrase “related group-based mindfulness interventions” and the term “mindfulness-related interventions” seem exactly the same. However, even if the author suggests the difference resides in the “group-based” aspect, many other interventions including MBSR and MBCT are also group-based. Furthermore, interventions in (1) are defined as training that foster mindfulness, whereas interventions in (2) are characterized as training that incorporate mindfulness as one component of the program. In reality, this is far from accurate, since MBSR and MBCT also involve multiple components including mindfulness. Therefore, after careful examination of the distinction made by the author, the major difference between (1) and (2) seems to be that the former has the term “mindfulness” in the name of the intervention, hence categorized as mindfulness-based interventions, but the latter does not. To provide a more thorough understanding, we outline below some of the similarities and differences in these interventions as discussed by leading researchers in the field (Kabat-Zinn, 1990; Segal et al., 2002; Davidson and Kabat-Zinn, 2004; Smith, 2004; Linehan, 2014; Tang et al., 2015; Hayes et al., 2016; Tang, 2017).

MBSR has multiple components including mindfulness, yoga exercise, body stretching, group discussion and other components in the program, just like (2) the mindfulness-related interventions mentioned above. For instance, MBSR was described as a “*program that focuses on learning how to mindfully attend to body sensations through the use of body scans, gentle stretching, and yoga mindfulness exercises, along with discussions and practices geared toward applying mindful awareness to daily life experiences, including dealing with stress”* (Creswell, 2017, p. 495). Therefore, it does not make sense to only characterize MBSR or MBCT as mindfulness intervention, but exclude other mindfulness interventions that simply do not have the term “mindfulness” in their names. Consistent with MBSR developer Kabat-Zinn's clarification in his book and later articles, there isn't a pure mindfulness program, and mindfulness intervention such as MBSR incorporates other techniques (Kabat-Zinn, 1990; Davidson et al., 2003; Davidson and Kabat-Zinn, 2004). Smith (2004) also pointed out that “MBSR system is an amalgam of mindfulness meditation, concentrative meditation, passive breathing exercises, yoga stretching, and other components.” Therefore, mindfulness intervention or training works through an integration of several techniques and components rather than a single technique - mindfulness. In the same vein, MBCT developers described the training as a program that draws from cognitive behavior therapy (CBT) and traditional mindfulness practices such as MBSR. By definition, MBCT is a psychological intervention for individuals at risk of depressive relapse (Segal et al., 2002). Clearly, MBCT also incorporates other trainings such as CBT into its program, and it does not make sense to suggest MBCT as a mindfulness intervention, but other similar programs (e.g., ACT, DBT, IBMT) without the term “mindfulness” are not. This clarification is crucial since the misunderstanding of what mindfulness interventions are will mislead the research community and general public on mindfulness and its application, and may create confusion or even bias for people who are interested in research and applied work in this field.

In reality, mindfulness is NOT just a concept or a term, instead it is a direct experience prior to one's conceptualization *per se*. Without any personal experience of mindfulness, one can only get partial reflection of that experience—perhaps like a blind man touching an elephant. Therefore, the name of a mindfulness meditation or intervention with or without the term “mindfulness” should not define the nature of the program. Instead, the exact components and instructions of mindfulness practice are the key to define the program. Moreover, we need to understand that mindfulness methods always include several components and there is no pure “mindfulness” with only a mindfulness component (Davidson and Kabat-Zinn, 2004; Smith, 2004; Tang, 2017). In the following section, we will provide examples to demonstrate two key components - body-based exercise and mind-based practice in mindfulness program (Tang, 2017).

## How mind-body practice works

Speaking more broadly, mind-body practices such as mindfulness meditation shares key components of body-based exercise and mind-based practice. Body-based exercise emphasizes specific posture and body control with a state of relaxed stillness or gentle movement, whereas mind-based practice focuses on self-control of attention, emotion, and thought in a mindful way (Tang et al., 2007, 2015; Hölzel et al., 2011; Tang, 2017). Most studies suggest that mind-based practice such as mindfulness plays a major role in enhancing the beneficial effects of training. However, recent evidence has shown that body-based exercise also has positive effects on cardiovascular system and central nervous system (CNS), and has been applied to promote health and alleviate chronic diseases (Kerr et al., 2013; Tang and Tang, 2015; Tang, 2017). Taken together, it remains unknown which components or ingredients of mind-body practice play a key role in producing training benefits.

In our series of randomized studies employing one form of mindfulness training - Integrative Body-Mind Training (IBMT), we selected relaxation training (RT) as an active control for two reasons: (1) our goal is not to compare which mindfulness training is the best, instead, we focus on IBMT as an intervention that influences the activity and connectivity of brain areas such as the anterior cingulate cortex which are parts of a self-control network. We assume that other forms of mindfulness training influencing similar brain regions will obtain similar results (Hölzel et al., 2011; Tang et al., 2015); (2) RT control has been widely used as an effective behavioral therapy. Research has shown that RT targets a series of somewhat different brain regions than IBMT. Brain regions affected by RT include sensory motor areas, prefrontal and parietal regions than IBMT (Tang et al., 2007, 2009; Tang, 2017). Since IBMT primarily involves 3 components—body relaxation, mental imagery and mindfulness training, whereas RT mostly involves 2 components—body relaxation (relaxing different muscle groups) and mental imagery (with eyes closed and in a sequential pattern, one concentrates on the sensation and feelings of warmth and heaviness of body), we assume that we could differentiate the effects of mindfulness component by subtracting RT from IBMT. Our previous results indicate that both IBMT and RT produce effects on brain and physiology even after a short-term practice (Tang et al., 2007, 2009, 2010, 2012, 2013, 2015), but they involve different brain areas and thus distinct mechanisms.

In general, the positive effects on behavior, physiology and brain following IBMT are greater than that of RT. At first, these results might seem to suggest that the mindfulness component alone is responsible for these significant differences. However, during our teaching experience when we worked with IBMT participants and applied only the mindfulness component, we could not achieve the similar effects as shown in our series of randomized studies (Tang, 2009), which suggests that IBMT combines different ingredients from the mind-body practices and it is the integration that produces the beneficial effects.

## Several possible reasons of why brief IBMT works effectively

First, IBMT integrates components of both body (including autonomic nervous system) and mind (including central nervous system), which together have shown to have overall positive effects on attention, emotion, and social behaviors (Tang et al., 2015). This combination and integration may amplify the training effects over the use of only one of these components.

Second, everybody can experience mindfulness moment in own life. A qualified coach or trainer can help each practitioner increase this experience and thus ensure that every practice session can achieve a mindfulness state (Tang et al., 2007; Tang, 2017). We view the role of the coach as extremely critical. The coach knows how to interact with the practitioners to achieve the desired mindfulness state. The trainers could well be a part of the effective ingredient in IBMT, and their role requires additional research.

Third, a soft music background integrates the instruction and occupies the practitioner's wandering mind through continuous sensory input, facilitating, and maintaining the mindfulness state. Many training techniques use music background to help beginners. Based on our series of research, we believe that the integration of various techniques into one easy-to-use training package may explain why IBMT is effective at such low dosage although we do not know which of these features is of greatest importance in obtaining changes.

In summary, in this *Opinion* piece, we clarify the misunderstanding of mindfulness-based interventions and summarize recent mindfulness findings to explore the question of how mind-body practice works. We propose that mind-body practice is a holistic system and works through the integration of different ingredients rather than separated components to achieve the beneficial effects.

## Author contributions

All authors listed, have made substantial, direct and intellectual contribution to the work, and approved it for publication.

### Conflict of interest statement

The authors declare that the research was conducted in the absence of any commercial or financial relationships that could be construed as a potential conflict of interest.
